# No one left behind: how are we doing in the roll-out of PrEP as part of combination HIV prevention?

**DOI:** 10.7448/IAS.19.7.21364

**Published:** 2016-10-18

**Authors:** Carlos F Cáceres, Linda-Gail Bekker, Peter Godfrey-Faussett

**Affiliations:** 1 Center for Interdisciplinary Studies in Sexuality, AIDS and Society, Universidad Peruana Cayetano Heredia, Lima, Peru; 2 Desmond Tutu HIV Foundation & Centre, Cape Town, South Africa; 3 United Nations Joint AIDS Programme, Geneva, Switzerland

**Keywords:** HIV, prevention, pre-exposure prophylaxis, programme implementation, demonstration studies, continuum of prevention and care, health financing

The 2015 World Health Organization (WHO) Guidelines on when to start antiretroviral (ARV) therapy and on pre-exposure prophylaxis (PrEP) for HIV [1] made two landmark recommendations: 1) the offer of ARV treatment to anybody diagnosed with HIV infection, regardless of CD4 levels (i.e. “test and offer”); and 2) the offer of oral HIV PrEP with a tenofovir-containing scheme to any person at substantial risk. As of mid-2016, many of the concerns about potential roll-out that emerged from clinical trials are quickly fading: real-life effectiveness has been demonstrated in 2015/2016 and qualified the role of some of the important undesired occurrences that had been anticipated such as low adherence and “risk compensation” leading to viral resistance and new infections [2]. Studies such as PROUD [3] have shown that at least men who have sex with men (MSM) who use PrEP are at significant risk (due to frequent condomless anal intercourse with casual partners of unknown serostatus), are highly adherent, and can benefit from PrEP effectiveness. PrEP uptake with high adherence in certain communities (e.g. the gay community in San Francisco) [4] is contributing to significant reductions in HIV infections in that city. In addition, studies assessing intermittent PrEP (e.g. the on-demand PrEP scheme used in Ipergay [5], and alternative schemes for MSM included in a Thai study) have also contributed exciting results [6].

On 8 June 2016, the UN General Assembly signed the political declaration on HIV and AIDS: on the fast-track to accelerate the fight against HIV and to end the AIDS epidemic by 2030 [7]. To fulfill the aspirations of the political declaration, UNAIDS has established a 2016–2021 strategy [8] with targets set in HIV prevention and care at the local and regional levels: an ambitious target of ensuring access to PrEP for three million people at substantial risk by 2020. This is based on reaching an overall estimate of 10% of populations at increased risk, namely key populations including MSM, transgender people, and sex workers as well as young women of reproductive age living in the most highly affected communities and people in serodiscordant partnerships where the viral load of the positive partner is not known to be reliably suppressed.

Despite recent progress and these aspirational goals, actual roll-out at a global scale is just beginning, and considerable challenges remain unmet. The planning and organization of demonstration studies beyond MSM in the United States and the UK has been slow, and implementation-relevant information for both the general population and specific key populations (e.g. female sex workers in generalized epidemic contexts, and MSM and transwomen in Asia and Latin America) is still lacking. While concerns about adherence and effectiveness (particularly among MSM) have abated, some other issues have emerged, including 1) the preferred ARV agent, although tenofovir–emtricitabine is the only licensed agent at present; 2) potential of less frequent dosing, cost and sustainability (as much in lower-middle income as in higher-income countries); and 3) a long list of implementation questions that vary by setting and target population. The latter may make monitoring of impact and outcome more complex.

NEMUS, in collaboration with UNAIDS, has followed its first supplement on PrEP beyond clinical trials [9] with this new collection of papers focused on PrEP roll-out, identifying barriers and solutions, again with a focus on regions and populations not considered before. In this editorial, we highlight the main messages of the papers included in this new series. Together with other tools that will be published soon, such as the upcoming WHO implementation guidelines, this publication will help to document progress and guide implementers through a rapidly changing field.

## Contributions included in this special issue

A paper by Cáceres *et al*. [9] discusses PrEP scale-up to date, including the observed levels of access and policy development, and elaborates on key emerging policy and research issues to consider for further roll-out, with a special focus on lower-middle income countries. While feasibility, acceptability and potential impact have been demonstrated, creative solutions will be needed to overcome challenges, which include operational and health systems barriers, drug cost and regulatory policies, health providers’ openness to prescribing PrEP to populations at substantial risk, demand creation and legal and human rights issues.

The contribution by McGillen *et al*. [10] examines what role PrEP should play in an optimal patterning of combination prevention in the complex and dynamic landscape of sub-Saharan Africa. The authors use a previously described mathematical model and focus on PrEP to explore how best to distribute PrEP within broader prevention resources. They propose that if current year-on-year financial contributions to prevention funding were to be maintained, an incidence benchmark, as per the new WHO guidelines, would serve as a reasonable way to determine where and to whom PrEP should be offered.

Also focusing on sub-Saharan Africa, Cowan *et al*. [11] state that at least 3 million individuals in Africa are likely to be eligible for PrEP according to WHO's criteria and that several African countries have already approved guidelines for PrEP for individuals at substantial risk of HIV as part of combination HIV prevention, but key questions remain about how to identify and deliver PrEP to those at greatest need. Overarching issues in each of the target populations remain, such as creating demand for PrEP, addressing supply-side issues and providing appropriate and tailored adherence support. Some key action areas identified included the normalization of HIV prevention to help demand creation; community level interventions involving opinion leaders as well as empowerment interventions for those at highest risk; access to quality services for all, including for stigmatized populations; and provision of adherence support that recognizes social and structural factors. They predict that combining interventions that build self-efficacy, empowerment and social cohesion is most likely to be effective in PrEP provision.

A paper by Zablotska-Manos *et al*. [12] discusses the progress towards PrEP implementation in the Asia/Pacific region. In this region, key PrEP implementation barriers include poor knowledge about and limited access to PrEP, weak or non-existent HIV prevention programmes for MSM and other key populations, high cost of PrEP, stigma and discrimination against key populations, and restrictive laws. While trials and implementation research is taking place only in Thailand and Australia, novel approaches to PrEP implementation have emerged (such as researcher-, facility- and community-led models of care, with PrEP services for fee and for free), and there is growing community interest in PrEP in the region. They conclude that countries in the Asia/Pacific region will benefit from adding PrEP to their prevention packages, but this will need investment.

Ravasi *et al*. [13] discuss the barriers encountered and potential solutions needed for a fair consideration of PrEP as part of combination HIV prevention strategies in Latin America. No Latin American country has yet implemented a PrEP programme, and so first steps including education of policy makers, programmatic guidance and costing models are still needed. Providers are not prescribing PrEP due to a lack of national policies and guidelines and lack of training. Encouragingly, key populations (MSM, transgender women (TW) and sex workers) have participated in demonstration projects and show high awareness and willingness to use PrEP, especially if accessible in the public sector for free or at affordable price. As in many regions, concerns about safety, adherence, effectiveness and risk compensation need to be addressed through targeted social communication strategies. The authors conclude that an alliance between policy makers, civil society and representatives from key populations, healthcare providers and researchers will kick-start implementation of PrEP demonstration projects and other steps needed for the successful roll-out of PrEP in Latin America.

The final regional paper by McCormack *et al*. [14] focuses on Europe, where PrEP is only available in France to date. In a region with considerable differences in health systems and government commitment to HIV prevention and care, the number of HIV infections is increasing, even in countries with free access to screening and treatment, among MSM and other key populations. As in many parts of the world, prevention funding is a fraction of care funding. Standards of care are generally good in Western Europe, but less satisfactory in Eastern Europe and central Asia, given limited national health budgets and diminishing foreign aid. Even in Western Europe's high-income countries, the cost of Truvada^®^ is a major barrier to PrEP implementation, together with inadequate health systems and a weakening civil society.

This special issue includes three papers focused on specific populations. One contribution by Sevelius *et al*. [15] focuses on TW, one of the key populations most affected by HIV, and discusses unique considerations for maximizing the impact of PrEP in this vulnerable population. They report that, to date, PrEP demonstration projects and clinical trials have largely excluded TW, or failed to include them in a meaningful way, limiting the ability of such studies to draw conclusions about TW's unique needs and devise strategies to meet them. The need for gender affirming services to facilitate the provision of PrEP to TW is critical. There is a need to engage trans communities, utilize trans-inclusive research and marketing strategies, and identify and/or train health care providers to provide gender affirming health care to trans women; in turn, health systems must consider and address TW's unique barriers and facilitators to uptake and adherence.

Hosek *et al*. [16] focus on the potential role of PrEP among young people and discuss data from the United States and South Africa on the use of oral PrEP for HIV prevention in adolescent minors, along with some of the implementation challenges and potential strategies to address those challenges. Adolescents and young adults less than 25 years of age in many geographical settings meet the definition of a key population in the HIV epidemic, with very high HIV incidence rates and limited access to prevention services. Completed and ongoing studies in the United States and South Africa among youth under age 18 should provide the safety data needed by the end of 2016 to contribute to licensure of Truvada^®^ as daily PrEP in adolescents. A number of general and unique challenges have arisen in this age group. Prime among these is adherence to daily medication, which is particularly challenging among younger populations, but other individual level barriers (e.g. limited familiarity with ARV-based prevention, stigma, product storage and social support) and structural challenges (e.g. healthcare financing for PrEP, clinician acceptability and comfort with PrEP delivery, and limited youth-friendly health services available) are also described.

In turn, Coleman and McLean [17] provide a discussion on the value of PrEP in HIV epidemics among people who use drugs (PWUD). While PWUD are at significant risk for HIV in many parts of the world and should be offered PrEP according to current recommendations, the actual feasibility of this strategy may require an enabling legal and policy environment for delivery of health services to those in need. The need to address structural barriers to services and human rights violations, and to improve access to comprehensive harm reduction programmes are of prime importance and may have higher value than a single focus on HIV prevention, is argued by the authors. If those conditions are not met, shifts in funding priorities, for example, to include PrEP, could threaten programme comprehensiveness, hence facing opposition by PWUD. Nevertheless, nuanced needs of sub-populations of PWUD and their partners must be explored. As for all key populations, the involvement of PWUD in shaping comprehensive services is vital and too often ignored.

Finally, Cairns and Race [18] present a community viewpoint, which reminds the readers that PrEP has been and continues to be an intervention causing controversy and debate between providers, advocates and potential users. Such controversies, they sustain, extend beyond access and can be related to contemporary tensions: medical risk versus benefit; trust versus distrust of healthcare interventions; and individual responsibility versus community cohesion. In that sense, PrEP might lead people to perceive a risk of losing control over any of those tensions. They close by suggesting that the development of greater community “ownership” of PrEP and concomitant improvements in the sense of individual agency over sexual risk might reduce the insecurities derived from those tensions and facilitate a more neutral uptake of this strategy.

Given the need presented in this series and the promise and potential of the clinical trials and recommendations from WHO, it is hoped that we will see an impressive expansion of combination prevention including PrEP in the next 2–5 years. With just 14 short years before the UNAIDS target to end AIDS by 2030, unless we urgently, actively and extensively deploy all of the effective interventions at our disposal, this goal will slip away from our grasp. No one should be left behind [19].
